# A Review of the Clinical Pharmacokinetics of Polymyxin B

**DOI:** 10.3390/antibiotics8010031

**Published:** 2019-03-22

**Authors:** Sean N. Avedissian, Jiajun Liu, Nathaniel J. Rhodes, Andrew Lee, Gwendolyn M. Pais, Alan R. Hauser, Marc H. Scheetz

**Affiliations:** 1 Department of Pharmacy Practice, Chicago College of Pharmacy, Midwestern University, Downers Grove, IL 60515, USA; savedi@midwestern.edu (S.N.A.); jliu@midwestern.edu (J.L.); nrhode@midwestern.edu (N.J.R.); gpais@midwestern.edu (G.M.P.); 2 Pharmacometrics Center of Excellence, Midwestern University Downers Grove, IL 60515, USA; 3 Department of Pharmacy, Northwestern Memorial Hospital, Chicago, IL 60611, USA; 4 Departments of Chemical and Biological Engineering, Northwestern University, Evanston, IL 60208, USA; alee2@midwestern.edu; 5 Departments of Microbiology-Immunology and Medicine, Northwestern University, Feinberg School of Medicine, Chicago, IL 60611, USA; ahauser@northwestern.edu; 6 College of Graduate Studies, Department of Pharmacology, Midwestern University, Downers Grove, IL 60515, USA

**Keywords:** polymyxin B, pharmacokinetics

## Abstract

Polymyxin B remains an antibiotic of last resort because of its toxicities. Although newer therapies are becoming available, it is anticipated that resistance to these agents will continue to emerge, and understanding the safest and most efficacious manner to deliver polymyxin B will remain highly important. Recent data have demonstrated that polymyxin B may be less nephrotoxic than colistin. Pharmacokinetically, polymyxin B is primarily eliminated via non-renal pathways, and most do not recommend adjusting the dose for renal impairment. However, some recent studies suggest a weak relationship between polymyxin B clearance and patient creatinine clearance. This review article will describe the clinical pharmacokinetics of polymyxin B and address relevant issues in chemistry and assays available.

## 1. Introduction

Infections caused by antibiotic-resistant Gram-negative bacilli are characterized by high morbidity and mortality [1]. The emergence of multi-drug resistance among Gram-negative pathogens has led to the revival in the use of polymyxins as antibiotics of “last resort.” Both colistin (polymyxin E) and polymyxin B were approved for clinical use in the 1950s, but severe nephrotoxicity and neurotoxicity limited their use by the early 1970s [2,3,4,5,6,7,8,9]. The polymyxins were approved before the requirement for rigorous pharmacokinetic (PK) data; thus dosing information in the approved product labelling is sparse [10]. Polymyxin dosing is challenged by variability in exposure profiles and a narrow therapeutic window needed to achieve efficacy while avoiding toxicity. However, of the two systemically active polymyxins (i.e., colistin and polymyxin B), polymyxin B displays less pharmacokinetic variability owing to the fact that it is administered intravenously in its active form [11]. Additionally, clinical studies suggest that polymyxin B is less nephrotoxic than colistin [12]. For these reasons, polymyxin B has become the predominant polymyxin used in many centers.

Polymyxin B remains very active against many multi-drug resistant organisms (e.g., *Pseudomonas aeruginosa*). As such, clinicians often rely on polymyxin B as a treatment alternative when other agents are contraindicated because of antimicrobial resistance. Despite the renewed interest in polymyxin B, optimal dosing strategies remain unclear. The purpose of this review is to describe the clinical pharmacokinetics of polymyxin B and address relevant issues in chemistry and assays available.

## 2. Chemistry

**Figure 1:**
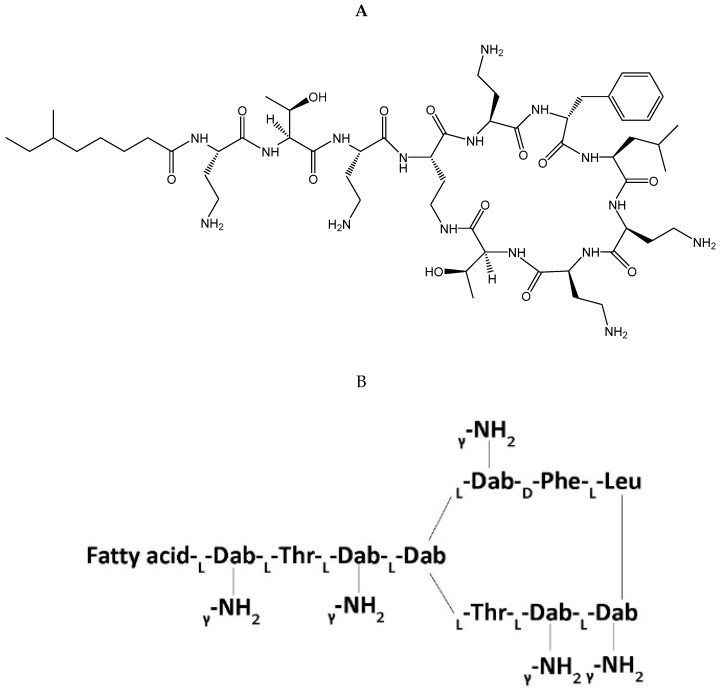
Stereochemical formula (**A**) and general molecular structure (**B**) of polymyxin B. Abbreviations: Fatty acid = 6-methyloctanoic acid for polymyxin B1, 6-methylheptanoic acid for B2, octanoic acid for B3, and heptanoic acid for B4, Dab = diaminobutyric acid, Thr = threonine, Phe = phenylalanine, Leu = leucine.

Polymyxin B is a cationic polypeptide antibiotic obtained from the fermentation products of the bacterium *Paenibacillus polymyxa* [11]. Polymyxin B’s core structure (Figure 1) consists of a polycationic peptide ring and a tripeptide side chain with a fatty acid tail [13]. Polymyxin B is a mixture of four polymyxin components (PB1, PB2, PB3, and PB4) with its major components consisting of polymyxin B1 (PB1), which contains the fatty-acyl group (S)-6-methyloctanoyl, and polymyxin B2 (PB2), which contains the fatty-acyl group 6-methylheptanoyl; however, proportions of each major component can differ depending on the clinical product manufacturer [14]. For clinical use, polymyxin B is administered intravascularly, intrathecally, aerosolized, or topically as polymyxin B sulfate [15]. It is not used orally due to poor bioavailability. Assays for pharmacokinetic application often are based on the major polymyxin subcomponents (i.e., PB1 and PB2), which comprise approximately 85% of total polymyxin B [16]. Sulfomethylated derivatives were developed to reduce the nephrotoxicity of polymyxins [17], but this was only carried forward for colistin as colistin methanesulfonate, which has been reviewed extensively elsewhere [18]. The sulfomethylated preparations for polymyxin B have not been developed clinically, presumably because of a lack of intrinsic activity [17].

## 3. Assay Methods for Drug Quantification

Quantification of polymyxins via high-performance liquid chromatography (HPLC) is difficult because of their low UV absorption, limited native fluorescence, and overlapping chromatographic profiles of the components [19]. This is further complicated by batch-to-batch differences in the ratio of PB1 and PB2 that can exist. Current methods for polymyxin B quantification favor the combination of liquid chromatography with mass spectrophotometry, including liquid chromatography tandem mass spectrometry (LC-MS/MS) and ultra-performance liquid chromatography tandem mass spectrometry (UPLC-MS/MS), over conventional bioassays and HPLC [11]. This is primarily due to the superior sensitivity, specificity, and accuracy of mass-spectrophotometry-based methods [20]. Multiple validated mass spectrometry methods for drug quantification for polymyxin B have now been published and are available for application in plasma and serum (human and/or rodent), epithelial lining fluid (mouse), and bacterial growth media [20,21,22,23,24,25,26]. A summarized list of validated methods can be found in Table 1. Enzyme-linked immunosorbent assay methods have also been developed for human serum, mouse plasma, and mouse kidney tissue [27,28,29]; however, they have not yet gained popularity for clinical PK application. 

## 4. Pharmacokinetics

With clinically administered doses, mean polymyxin B maximum serum concentration (Cmax) at steady-state ranges from ~2–14 mcg/mL, and polymyxin B half-life is ~9–11.5 hours [31,32,33]. Polymyxin B’s proposed mechanisms of drug elimination involve both renal (via renal tubular reabsorption) and non-renal pathways. Data suggest that polymyxin B preferentially accumulates in renal tissue in rodent models, and this may account for the apparent clearance [27,34]. Further, multiple studies in both animals and humans have shown that urinary recovery of polymyxin B is low (<5%), suggesting a selective uptake and residence process in renal cells [34,35,36,37]. Despite the fact that the mechanism of the non-renal clearance of polymyxin B is not fully elucidated, it is proposed as the predominant clearance pathway for polymyxin B [36]. Biliary excretion has been suggested, as all four components of PB have been detected in bile [37]. Further studies examining the non-renal routes of polymyxin B elimination are warranted.

### 4.1. Mechanisms for Nephrotoxicity

Studies investigating the mechanism for polymyxin B nephrotoxicity are ongoing. Studies conducted in in vitro and in vivo models have shown that polymyxin B has the potential to be toxic to renal tubular cells [38]. The cellular mechanisms proposed for nephrotoxicity include oxidative stress, apoptosis, cell cycle arrest, and autophagy. Several studies have suggested a role for megalin in nephrotoxicity mediated by polymyxin B [39,40]. Briefly, megalin is a member of the low-density-lipoprotein-related protein 2 receptor gene family that is predominately expressed in the microvilli of renal proximal tubular cells [41]. It functions as an endocytic receptor and is responsible for the internalization and uptake of a wide variety of endogenous molecules. Polybasic drugs such as polymyxin B have a high-binding affinity for megalin [40]. The current hypothesis for polymyxin-induced nephrotoxicity, which is supported by cellular studies [39], is that polymyxin B accumulates in the proximal tubule after apical megalin-mediated uptake (of polymyxin B from the luminal space). Cellular accumulation is then thought to drive cell death and nephrotoxicity. These findings were followed up in 2017 with an animal model utilizing megalin-shedding rats [42]. The authors showed that the megalin-shedding rats had renal tissue exposures attenuated by approximately 40% compared to control animals. While megalin is intriguing, the full cause of renal toxicity is complicated. The exact mechanism is not yet completely understood, and more studies are required.

### 4.2. Population Pharmacokinetics (PK) Models

Reported population PK data for polymyxin B are limited. Contemporary polymyxin B dosing recommendations have largely come from studies that focused on population PK [35] and on understanding free-fractions and urinary clearance [36]. More recently, additional reports have emerged and provide further understanding in the variability of polymyxin B in various patient populations, thus allowing some exploration of clinical variable relationships affecting polymyxin B disposition [32,33,42,43]. A complete list of population polymyxin B PK models with patient populations studied and estimates of PK parameters can be found in Table 2. Briefly, researchers have modeled polymyxin B with 1- and/or 2-compartment models and various fitting strategies. The PB1 component has been modeled singly [44], but many recent studies now assay various subcomponents of polymyxin B such as PB1 and PB2 [32,33,35,45]. Representative modeling (e.g., PB1 modeling) is based on the idea that pharmacokinetic handling of the major components of polymyxin B is similar [46,47]. The largest PK patient population study to date consisted of 52 adult patients [33]. Specific populations examined in these studies include the acutely-ill, critically-ill, those with normal/insufficient renal function, cystic fibrosis (CF) patients, as well as individuals with multidrug-resistant Gram-negative bacterial infections. 

### 4.3. PK Parameter Estimates

Overall, the PK parameter estimates were similar from studies that utilized comparable compartmental models. Briefly, the studies that employed a 1-compartment model all found consistent estimates for clearance (CL) at 2.4, 2.37, and 2.5 L/h [32,43,45]. Similarly for volume of distribution (V1), estimates were 47.2, 34.4, and 34.3 L. For the 2-compartment models, CL values were 0.0276 L/h/kg, 2.5 L/h (normal renal function), 2.0 L/h (renal insufficiency), and 2.63 L/h [33,35,44]. For V1, estimates were 0.0929 L/kg and 33.77 L, and for V2 estimates were 0.330 L/kg and 78.20 L. As only one study utilized a 2-compartment model with polymyxin B clearance described as a Hill function, the PK parameter estimates from that model can be found in Table 2. It is important to note that while central tendency estimates were similar, variability in population pharmacokinetic models was high with CV% often >30% for the population PK parameter estimates. Thus, there is a role for patient-specific dosing via adaptive feedback and control as later described.

### 4.4. Clinical Variables Affecting PK

The impact of clinical variables on polymyxin B PK was explored in the studies mentioned above (Table 2); however, the findings were not always consistent. Specifically, the impact of total body weight (TBW) and creatinine clearance (CrCL) on polymyxin B clearance warrants further investigation. The impact of TBW was examined by Sandri and colleagues [35], who found a lower between-subject variability when TBW was linearly scaled to volume of distribution (V1) and CL. They also examined the potential relationship of polymyxin B CL (scaled and unscaled) with CrCL, APACHE II score, sex, age, and serum ablumin concentaton. These investigations did not reveal significant relationships [35]. Miglis and colleagues [33] analyzed the relationship between TBW and both volume of distribution and clearance. This study suggested a weak relationship, however recommended a first dose load to be weight-based to meet early area under the curve (AUC)-based pharamcodynamic targets [33]. Subsequent doses were suggested to be weight-independent to avoid toxicity. With the same patient population, Kubin and colleagues [45] also investigated the relationship between TBW and polymyxin B CL and found the variable-adjusted model did not produce overall model improvement [45].

With regard to creatinine clearance, Avedissian and colleagues [43] examined clinical variable predictors of PK in nine CF patients and found a potential relationship between patient-estimated CrCL and polymyxin B CL [43]. Polymyxin B CL was best explained according to patient CrCL via a Hill function. It is unclear if polymyxin B CL increases at higher CrCL, if polymyxin B CL is different in CF patients, or if the finding is artifact. A study in 2017 by Thamlikitkul and colleagues [48] specifically compared the polymyxin B CL and exposure estimates between normal renal function (CrCL ≥ 80 mL/min) and renal insufficient patients (CrCL < 80 mL/min) [48]. After standardizing AUC for daily polymyxin B dose, exposures were found to be similar between the two groups (28.6 mg·h/L vs. 29.7 mg·h/L, *p* = 0.8). When comparing CL values between the two groups (2.5 L/h vs. 2.0 L/h, *p* = 0.06), the values did not statistically differ but the constrained power of the study might mean that this 25% absolute difference is relevant. A study by Manchandani and colleagues [32] also identified CrCL as a statistically significant variable of polymyxin B CL [32]. However, the relationship was not explored further. With three small studies demonstrating a borderline effect of CrCL on polymyxin B CL, the relationship warrants further investigation. 

Taken together, it appears that polymyxin B should be dosed in a weight-independent fashion after a potential loading dose. There are currently limited data for making polymyxin B renal adjustments when CrCL is within the standard physiologic range (i.e., ~140 mL/min or below). As the overall number of patients studied is still small (~191 patients), larger studies are needed to fully explore the impact of clinical variables on polymyxin B PK [49]. Such studies are currently underway (NCT02682355) [50].

### 4.5. Clinical Dosing Implications

A guideline for the optimal usage of polymyxins is now available [51] and reviews many of the studies cited here. Several additional studies were published after guideline review and attempted to address weight-based dosing for polymyxin B and the importance of creatinine clearance on polymyxin B clearance. Despite the renewed interest in polymyxin B for treatment of multidrug resistant Gram-negative bacteria, optimal dosing strategies remain unclear as they are largely based on population pharmacokinetics. Initial product labelling recommended dosing IV polymyxin B at 1.5–2.5 mg/kg/day divided into two daily doses [10]. For individuals with renal impairment, a dose of 1.5 mg/kg/day was suggested. The first major study to challenge this dosing was conducted by Sandri and colleagues [35]. These authors compared multiple different dosing strategies (i.e., loading dose ± different weight-based doses) via simulation and suggested that IV polymyxin B doses should be scaled by TBW and not adjusted for renal function [35]. Depending on the minimum inhibitory concentration (MIC) of the organism, they recommended that doses up to 3.0 mg/kg/day ± a loading dose be considered to reach the goal 24-hour area under the curve/minimum inhibitory concentration (AUC/MIC) of 20 mg·24 h/L (after adjusting for free fraction (f_U_) of polymyxin B = 0.42) for severe infections [35]. Miglis and colleagues [33] studied a separate population and simulated multiple weight-based dosing strategies (e.g., loading dose ± fixed dose vs. weight-based dose) to the target 24-hour AUC/MIC obtained from each dosing strategy [33]. They found that a regimen that included a loading dose of 2.5 mg/kg of TBW plus a fixed dose of 100 mg every 12 hours had the highest probability of achieving a 24-h AUC/MIC of ≥50 mg × 24 h/L (equivalent to ~20 mg·24 h/L after adjusting for the unbound fraction of polymyxin B) with the lowest likelihood of toxicity for all except those less than 50 kg. This was the first study to recommend a weight-independent maintenance dose (i.e., fixed dose) for polymyxin B. Avedissian and colleagues [43] studied a CF patient population and identified increased polymyxin B CL as a function of CrCL; however, the authors cautioned against translating this to using higher than standard doses in CF patients without further study [43]. The most recent study by Manchandani and colleagues [47] also did not find a relationship between weight and volume of distribution [32]. Thus, it is unclear what clinical variables can improve population models. Adaptive feedback and control have been suggested as a necessary standard for clinical polymyxin B dosing [49,52]. Effectively, this requires real-time assay of polymyxin B and application of an individualized approach (e.g., Bayesian maximum a posteriori probability approach). In short, a single concentration is measured from the patient and the most likely exposure profile is obtained. Clinicians would then use this information to create patient-specific (as opposed to population specific) dosing strategies.

## 5. Conclusions

With the ever-present threat of multidrug resistant Gram-negative bacteria, polymyxin B remains an important antibiotic agent, but safe and effective dosing strategies remain challenging. Multiple methods exist to quantify polymyxin B drug concentrations in various human biomatrices, but availability of these assays is limited. Thus, while patient-tailored dosing may be desired, most treatment continues according to population-based dosing models despite wide inter-patient variability. Future work is needed to clarify the importance of patient weight and renal function for the clearance of polymyxin B. Ultimately, adaptive feedback and control is likely needed to achieve the precise exposures necessary for efficacy and safety. 

## Figures and Tables

**Table 1:** Mass spectrometry assay methods for quantification of polymyxin B.

Study	Quantification Instrument/Method	Internal Standard	Precursor Ion → Product Ion Transitions (m/z)	Biomatrix	Solvents Utilized (Mobile Phases)
Cao et al. 2008 [25]	HPLC	N/A	PB1: 602.4, 401.9 PB2: 595.4, 397.2	Human plasma	Acetonitrile/tetrahydrofuran/ water (50:25:25, *v/v*)
Cheng et al. 2010 [23]	LC-MS/MS	Fibrinopeptide B (human)	PB1: 602.5 → 241.2 PB2: 595.6 → 227.5 PE1: 578.5 → 227.2 PE2: 585.6 → 241.3 Fibrinopeptide B: 786.3 → 187.3	Rat plasma	Acetonitrile with 0.1% FA, water with 0.1% FA
Thomas et al. 2012 [21]	LC-MS/MS	N/A	PB1: 602.6 → 241.1 PB2: 595.5 → 227.1	Human Plasma	Acetonitrile, water with 0.1% FA
He et al. 2013 [22]	UPLC-MS/MS	Carbutamide	PB1: 402 → 101 PB2: 397 → 101 PB3: 398 → 101 Ile-PB1: 402 → 101 Carbutamide: 272 → 74	Mouse Serum, ELF	Acetonitrile, water with 0.1% FA
Cheah et al. 2014 [24]	LCMS	Colistin in acetonitrile/water (50:50, *v/v*)	PB1: 401.85 PB2: 397.20 CA: 390.55 CB: 385.95	Bacterial growth media	Acetonitrile, water with 0.1% FA
Meng et al. 2016 [30]	LC-MS/MS	CB-182,753 (proprietary semi-synthetic cyclic peptide)	PB1: 602.6 → 241.2 PB2: 595.9 → 227.2 PB1-1: 602.6 → 241.2 CB-182,753: 614.4 → 532.6	Human plasma, urine	Acetonitrile with 1% FA in methanol (50:50), water with 0.1% FA, water/acetonitrile/methanol (10:45:45)
Covelli et al. 2017 [20]	LC-MS/MS	PE2 (i.e., Colistin B)	PB1: 402.3 → 100.9 PB2: 397.5 → 100.9 PE2: 386.2 → 100.9	Human and rat plasma	Acetonitrile/methanol (50:50) with 0.5% FA and 0.01 TFA, water with 0.5% FA and 0.01% TFA
Hee et al. 2017 [26]	LC-MS/MS	N/A	PB1: 602.6 → 101.2 602.6 → 241.2 PB2: 595.6 → 101.2 595.6 → 227.2 PB3: 595.6 → 101.2 595.6 → 227.2 Ile-PB1: 602.6 → 101.2 602.6 → 241.2	Human plasma	90% Acetonitrile with 0.1FA, water with 0.1% FA and 0.1% TCA

Abbreviations: PB1 = polymyxin B1, PB1-1 = polymyxin B1-1 (component of PB), PB2 = polymyxin B2, PE2 = polymyxin E2, CA = colistin A, CB = colistin B, ELF = epithelial lining fluid, FA = formic acid, TFA = trifluoroacetic acid.

**Table 2:** Summary of population pharmacokinetic (PK) studies for polymyxin B.

Study	Program Utilized for PK Modeling	Compartmental Model	Number of Patients in the Model	Total Number of Polymyxin B Serum Levels Included in Model	Utilized Simulations	Patient Population Studied	Population Estimates of PK Parameters (means)	CV% of PK parameters (%)
Kwa et al. 2008 [44]	NPEM	1 compartment	9	19	No	MDRGNO	Ke (h^−1^) = 0.051 CL (L/h) = 2.4 V1 (L) = 47.2 T ½ (h) = 13.6	Ke^#^: 78.4 CL: n/a V1^#^: 60.8 T ½: n/a
Zavascki et al. 2008 [36]	PK Functions for Microsoft Excel	Non-compartmental analysis (PK Functions for Microsoft Excel)	8	55*	No	Critically Ill	CL (mL/min/kg) = 0.50 V1 (mL/kg) = 137.8	CL^#^: 40.5 V1^#^: 36.6
Sandri et al. 2013 [35]	S-ADAPT	2 compartments	24	~192*	Yes	Critically Ill	CL (L/h/kg) = 0.0276 V1 (L/kg) = 0.0939 V2 (L/kg) = 0.330 CL_ic_ (L/h/Kg) = 0.146	CL: 32.4 V1: 73.3 V2: 70.1 CL_ic_: 50.4
Thamlikitkul et al. 2017 [48]	ADAPT 5	2 Approaches 1 compartment 2 compartments	19	~76*	No	Normal renal function and renal insufficiency	Only 2 compartment estimates shown CL_NR_ (L/h) = 2.5 CL_RI_ (L/h) = 2.0 (other PK parameters not listed)	CL_NR_^#^:16 CL_RI_^#^: 30
Miglis et al. 2018 [33]	PMetrics	2 compartments	52	156	Yes	Acutely Ill	CL (L/h) = 2.63 V1 (L) = 33.77 V2 (L) = 78.20 Q (L/h) = 2.32	CL: 53.6 V1: 45.0 V2: 47.9 Q: 57.4
Kubin et al. 2018 [45]	Monolix	1 compartment	43	134	Yes	Acutely Ill	CL (L/h) = 2.37 V1 (L) = 34.4	CL^#^: 41.5 V1^#^: 40.0
Avedissian et al. 2018 [43]	PMetrics	2 compartments with a Hill function	9	31	Yes	CF	CL_max_ (L/h) = 8.65 V1 (L) = 20.39 V2 (L) = 174.69 CL_nr_ (L/h) = 0.07 Q (L/h) = 2.85 CrCL_50_ (mL/min) = 141.24 H = 7.84	CL_max_: 35.7 V1: 20.6 V2: 20.6 CL_nr_: 31.4 Q: 85.1 CrCL_50_: 25.6 H: 29.4
Manchandani et al. 2018 [32]	ADAPT 5	1 compartment	35	139	Yes	Acutely Ill	CL (L/h) = 2.5 V1 (L) = 34.3 T ½ (h) = 10.1	CL: 43.8 V1: 47.8 T ½: n/a

Abbreviations: CL = clearance, V1 = volume in central compartment, V2 = volume in peripheral compartment, CL_ic_ = intercompartmental clearance, Q = intercompartment flow, CF = cystic fibrosis, H= Hill coefficient, CL_max_ = maximum polymyxin B clearance, CrCL_50_ = creatinine clearance at the 50% maximal rate of polymyxin B clearance, CL_nr_ = non-renal clearance, negative, MDRGNO = multidrug resistance Gram-negative bacterial organisms, CL_NR_ = clearance in normal renal function group, CL_RI_ = clearance in renal insufficient group, CV = coefficient of variation (i.e., between-subject variability).* Estimated by amount of levels per patient mentioned in methods given number of samples not listed in study. #CV% not reported in study and calculated from means and standard deviations reported.
